# How to use machine learning and fuzzy cognitive maps to test hypothetical scenarios in health behavior change interventions: a case study on fruit intake

**DOI:** 10.1186/s12889-023-17367-z

**Published:** 2023-12-11

**Authors:** Samvel Mkhitaryan, Philippe J. Giabbanelli, Maciej K. Wozniak, Nanne K. de Vries, Anke Oenema, Rik Crutzen

**Affiliations:** 1 Department of Health Promotion, CAPHRI, Maastricht University, P.O. Box 616, 6200 MD Maastricht, The Netherlands; 2 Department of Computer Science & Software Engineering, Miami University, Oxford, OH USA; 3 KTH Royal Institute of Technology: Stockholm, Stockholm, SE Sweden

**Keywords:** Machine learning, Genetic algorithms, Fuzzy cognitive maps, Complex interventions

## Abstract

**Background:**

Intervention planners use logic models to design evidence-based health behavior interventions. Logic models that capture the complexity of health behavior necessitate additional computational techniques to inform decisions with respect to the design of interventions.

**Objective:**

Using empirical data from a real intervention, the present paper demonstrates how machine learning can be used together with fuzzy cognitive maps to assist in designing health behavior change interventions.

**Methods:**

A modified Real Coded Genetic algorithm was applied on longitudinal data from a real intervention study. The dataset contained information about 15 determinants of fruit intake among 257 adults in the Netherlands. Fuzzy cognitive maps were used to analyze the effect of two hypothetical intervention scenarios designed by domain experts.

**Results:**

Simulations showed that the specified hypothetical interventions would have small impact on fruit intake. The results are consistent with the empirical evidence used in this paper.

**Conclusions:**

Machine learning together with fuzzy cognitive maps can assist in building health behavior interventions with complex logic models. The testing of hypothetical scenarios may help interventionists finetune the intervention components thus increasing their potential effectiveness.

## Background

**Fig. 1:**
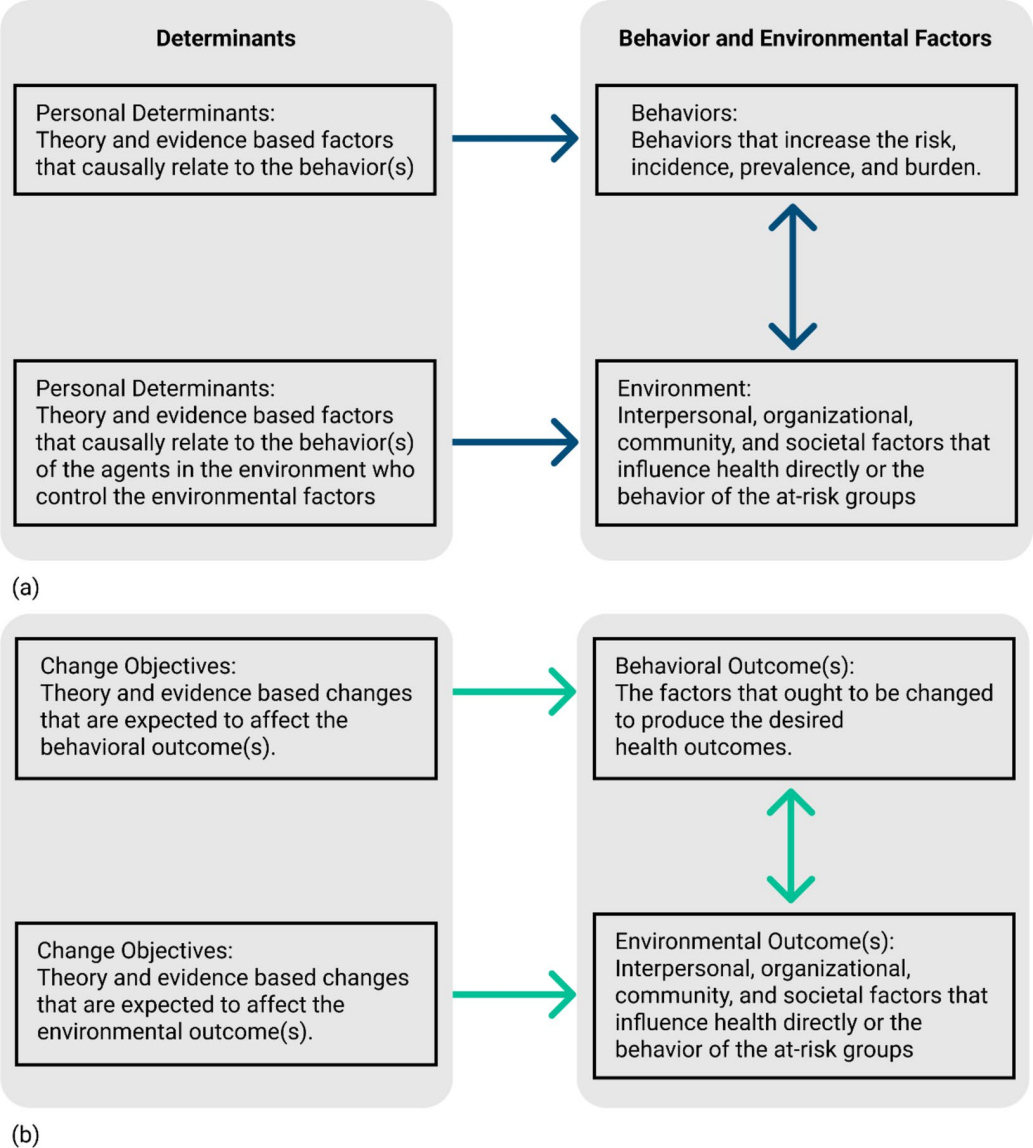
(**a**) A simple logic model based on PRECEDE model and (**b**) a logic model of change [1]

Many planning frameworks to guide the development of evidence-based health behavior change interventions start with conceptualizing the problem at hand. Intervention planners often do so by building a *logic model of the problem* [1, 2]. The logic model of the problem can make use of previously tested theoretical frameworks, conceptual frameworks, empirical evidence, or a combination of the three [3–6]. In short, a logic model of the problem constitutes a causal-path diagram that represents how a given problem has arisen and is being sustained. In the subsequent stages of the intervention development, the logic model of the problem is translated into a *logic model of change* (a.k.a. theory of change) which illustrates how specific intervention components are expected to result in a desired solution to the given problem (see Fig. 1). The logic model of the problem and the logic model of change are crucial for designing intervention components and properly defining research questions and measures both in process and outcome evaluation studies [1].

To build a comprehensive logic model of a given problem, intervention planners ought to meaningfully integrate evidence of various types from multiple sources. Types of evidence include both quantitative and qualitative data that come from expert panels, grassroot surveys, systematic reviews and meta-analysis, and theories related to behavior and behavior change [1].

Logic models commonly used in health behavior interventions (e.g., often simplified as chains of lists) are not limited to a certain type of evidence: they can integrate both qualitative and quantitative data as needed. Such flexibility is possible because the causal paths in these models are often defined at higher levels of abstraction (e.g., personal, behavioral, environmental) and do not specify either the nature or strength of the causes (Fig. 1a). This high-level specification of causal paths also makes such models intuitive to interpret as we can visually trace the effect of the intervention on the problem node. Nonetheless, these relatively simple logic models run the risk of *oversimplifying* the problem of interest by missing out on details that are important to plan, conduct, and evaluate an intervention.

**Fig. 2:**
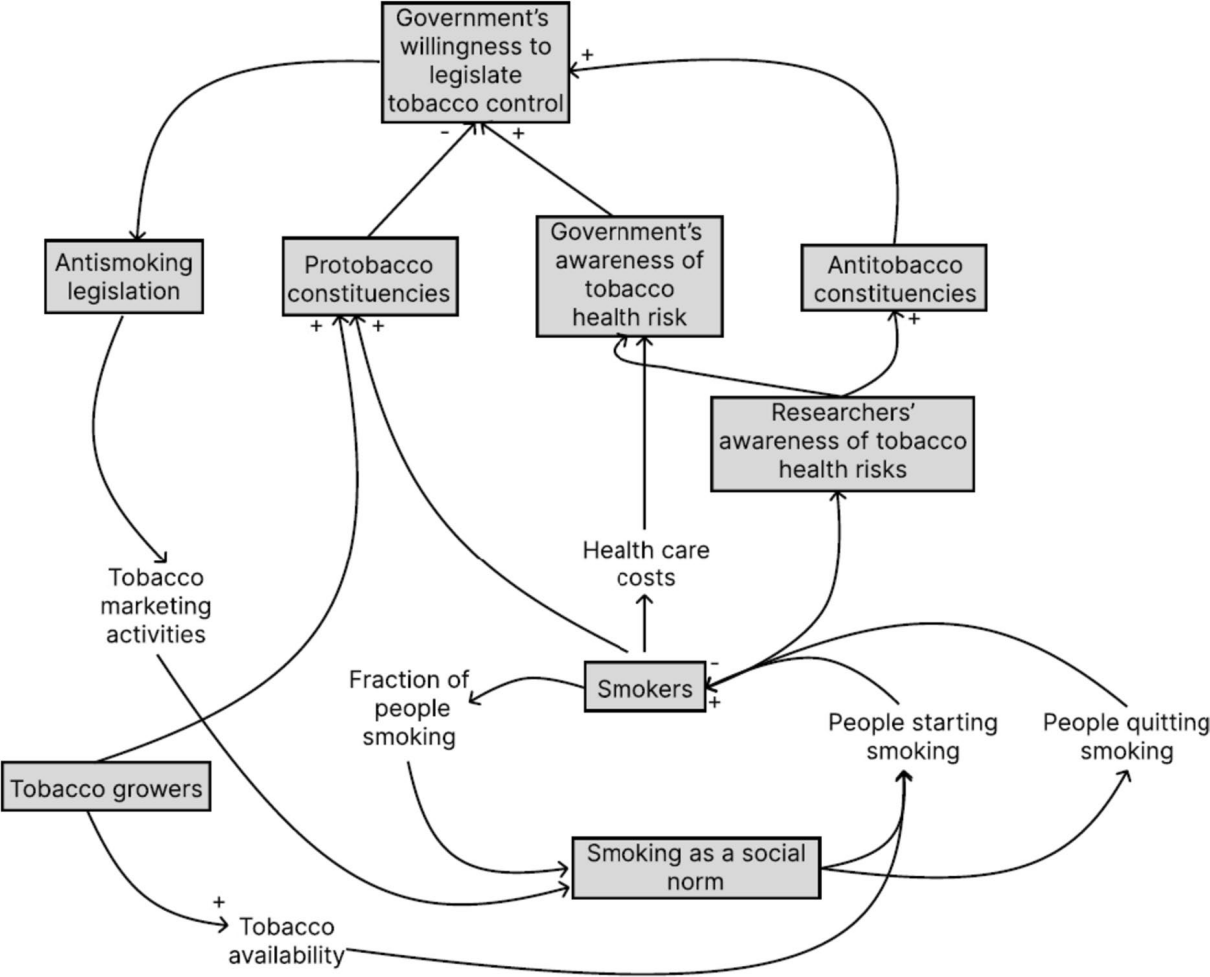
An excerpt of a more complex logic model of government control of tobacco [7]

Alternatively, we can incorporate information on lower levels (e.g., specific attitudes and beliefs) into a logic model, which requires knowledge of the nature and strength of causal paths (Fig. 2) [7]. Although this is appealing in terms of representing more information about the problem, this also faces two major challenges. Firstly, the struggle to integrate heterogeneous datasets within a single analytical framework. This integration is a necessity for practitioners who utilize survey data from primary sources, various types of effect-size metrics from secondary sources (e.g., Cohen’s *d*, odds ratios, correlation coefficients), qualitative evidence and expert opinions (both from primary and secondary sources). Secondly, because such frameworks permit more complex causal structures within a set of determinants (e.g., feedback loops instead of merely presenting chains of lists), it becomes difficult to estimate how a given intervention will unfold without additional computational techniques. For example, unlike in systems with simpler structure (e.g., a tree), in systems with causal loops the effect of an intervention will keep cycling between certain components which may inhibit the effect of an intervention or reinforce it.

Thus, effective evidence- and theory-based interventions require a framework that permits systematic integration of evidence of different types (qualitative and quantitative), accounts for the complex structure and functioning of interactions between the identified determinants, and provides computational techniques to assist decision making (i.e., testing of intervention scenarios). In our previous work, we discussed how a hybrid approach of Fuzzy Cognitive Maps (FCM) and machine learning could theoretically meet the aforementioned requirements [8]. In the present work, we provide empirical evidence that this potential can be realized, using a case study from a real-world intervention.

## Case study setting

**Fig. 3:**
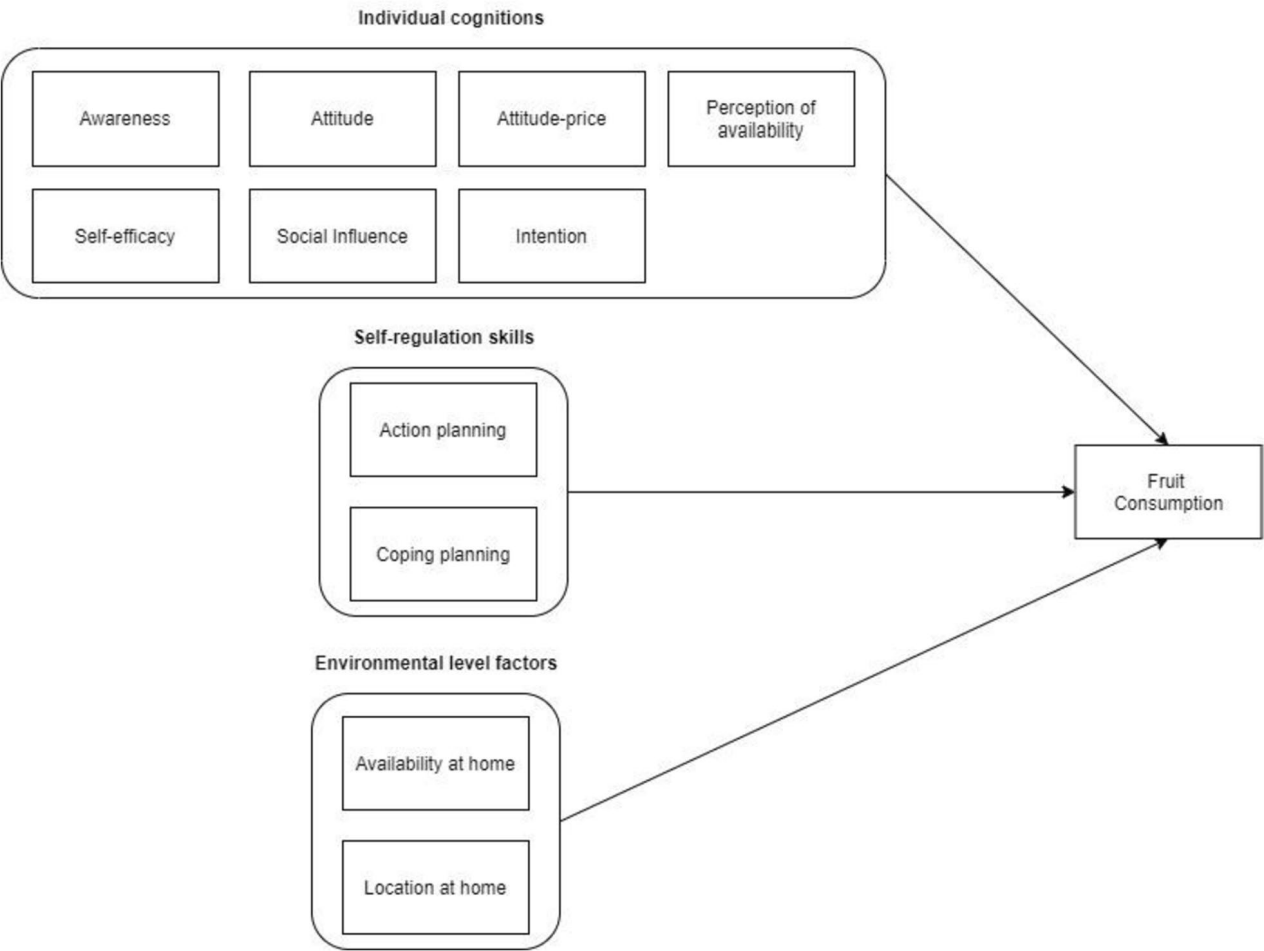
Logic model of fruit intake among adults in the Netherlands (adopted based on the conceptual model from Springvloet et al*.*, [9])

The intervention we selected for this case study is directed towards promoting healthy eating among adults in the Netherlands [9]. For the purposes of this study, we focus on fruit intake because the average habitual daily fruit consumption of Dutch adults was estimated to be below recommendation (117.4 compared to the recommended 200 g) [9–11]. Springvloet et al*.* [9] identified a broad list of personal and environmental determinants (Table 1) of unhealthy eating through a comprehensive review of the empirical literature and by surveying relevant theories (e.g., self-regulation theory, the precaution adoption process model, the theory of planned behavior and social cognitive theory). The determinants were grouped into three domains: i) individual cognition, ii) self-regulation skills, and iii) environmental level factors. The individual cognition domain included 1) awareness of the fruit intake; 2) attitude towards fruit intake; 3) attitude towards price related to fruit intake; 4) perception of availability; 5) self-efficacy; 6) social influence; and 7) intention. The self-regulation skills included action planning and coping planning. Lastly, the environmental level factors included the availability and the location of fruits at home. These determinants can be represented in a logic model of fruit intake (Fig. 3).

**Table 1:** Determinants of fruit intake and their operationalizations [12]

#	Construct	Operationalization
1	Awareness	One's awareness of the number of fruits s/he thinks s/he eats?
2	Attitude	One's belief that eating 2 servings of fruits daily is healthy
3	Attitude Price	One's belief that eating 2 servings of fruits daily is expensive
4	Self-efficacy	One's belief that s/he can eat more fruit per day in the next six months if s/he really wants to?
5		One's belief about the extent to which it is difficult to eat more fruit in the next six months?
6	Social-influence	One's belief that most people who are important to her/him think s/he should eat two pieces of fruit per day
7		One's belief that most people who are important to him/her consume two pieces of fruit per day
8	Intention	One's intention to eat two pieces of fruit per day?
9	Action-planning	One has a clear plan for when s/he is going to eat more fruit
10		One has a clear plan for which fruit s/he is going to eat more/less
11		One has a clear plan for how many fruits s/he is going to eat more/less
12	Copying planning	One has a clear plan for what s/he is going to do when something interferes with his/her plans to eat more fruit
13		One has a clear plan for what s/he is going to do in situations in which it is difficult to eat more fruit
14	Perception of availability at home	How often does one have fruit products available at home?
15	Visibility at home	Visibility of fruits at home

The model in Fig. 3 reads from left to right, includes a few causal paths, and does not include complex structures (feedback cycles). The intervention assumes that the change in the determinants will lead to an expected change in the fruit intake. Two important observations can be made about this logic model, which are representative of many available logic models. Firstly, the determinants are grouped into larger theoretical/conceptual domains (i.e., individual cognitions, self-regulation skills, environmental level factors) and the causal relationships are only considered between these larger domains and the problem node (fruit intake). Secondly, no consideration is given to the (possible) relationships between the determinants at the lower levels (i.e., the determinants within these theoretical/conceptual domains). For instance, knowledge, awareness and attitudes are interlinked. Individuals’ attitudes and beliefs depend at least partly on the information available to them. Similarly, the attitudes and beliefs will determine what type of information a person will search for (i.e., confirmation bias) [13]. This creates a reinforcing feedback process between knowledge, awareness, attitudes and beliefs. Such reinforcing processes reflect the tendency of an individual to align the elements of their cognition to avoid or minimize cognitive dissonance and the associated mental and physical discomfort [14–17]. The reinforcing processes are not only hypothesized to exist between the psychological determinants but also between the determinants within the domains of person, behavior and the environment in general (i.e., triadic reciprocal relationships in Bandura’s social cognitive theory) [4, 18].

In the subsequent section, we provide a brief introduction to FCMs, describe the machine learning algorithm to build an FCM model based on longitudinal data from this case study and use computer simulations to assist decision making (i.e., test hypothetical intervention scenarios).

## Methods

FCMs represent a system of interdependent components in a weighted signed directed graph. The nodes in the graph represent the factors included in the system (e.g., determinants) and the weighted signed directed arcs represent the causal relationships between the causal factors. The FCMs are equipped with simulation capabilities which permit exploration of the effects of various hypothetical scenarios. To construct an FCM one needs information on the factors that are relevant for a given case and the causal relationships between these factors (i.e., direction and strength of the causal relationships) [8].

For this intervention case, we already had a comprehensive list of determinants of the fruit intake, as described in Springvloet et al*.* [9]. To construct the FCM for this case study, we needed to add the potential causal paths between these determinants. For this, we used the baseline measurements of each determinant among the group of participants who did not receive any intervention in between the measurement time points (i.e., control group). The dataset contained 257 observations on the 15 determinants and the outcome variable (fruit intake) (see Table 1). The data was collected in the period of March 2012 and December 2013. The details on the socio-demographic profiles of the study participants can be found at [12]. The determinants in the domain of individual cognition and self-regulation skills were assessed on a 5-point Likert scale whereas the environmental determinants were assessed on a nominal scale. The fruit intake variable was created as a composite score based on multiple indicators (more information about the survey can be found in Springvloet et al*.* [12]).

**Fig. 4:**
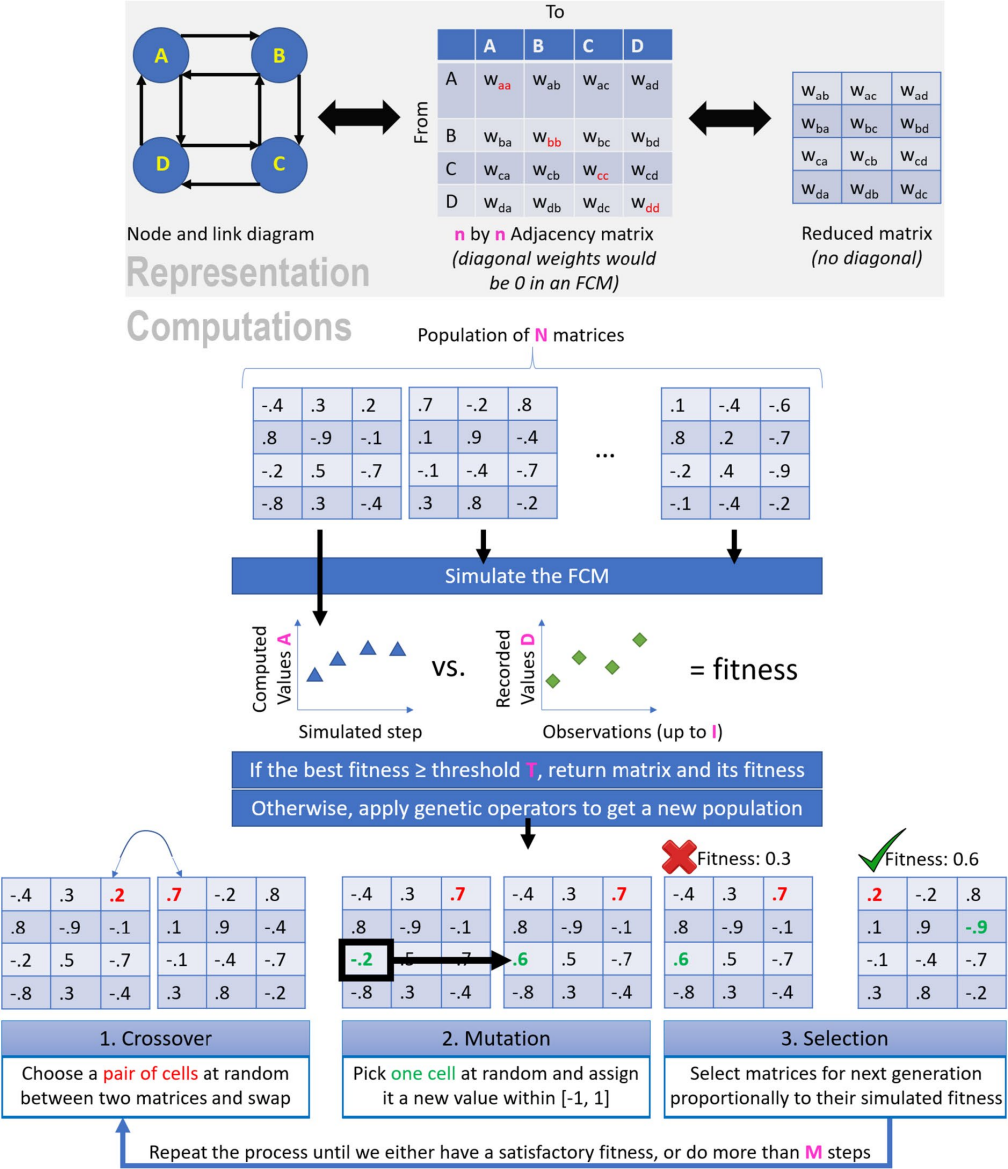
Representation (top) and computations involved in optimizing an FCM using the Genetic Algorithm approach summarized in Algorithm 1

In an FCM created via Machine Learning, whenever longitudinal data on the determinants is available, we can use data-driven approaches (e.g., Nonlinear Hebbian Learning algorithm, Real Coded Genetic Algorithm (RCGA)) to identify the potential causal links between the determinants [19–21]. The RCGA algorithm generates FCMs based on longitudinal data and requires no expert intervention in the learning process. In this study, we use a modified version of the legacy RCGA algorithm proposed by Stach [21]; our method is summarized at a high-level by Algorithm 1, which is detailed in a technical publication [22] and illustrated in Fig. 4. The algorithm is able to reproduce the trajectory of an individual's behavior because we seek an optimal fitness at *every* step, instead of optimizing only the final stage as was done in prior work (Fig. 4).

**Figure:**
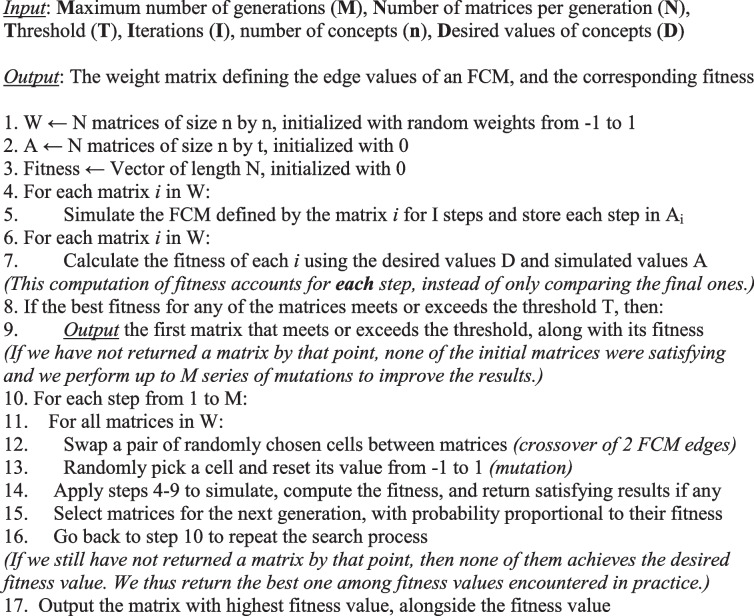
**Algorithm 1. **Pseudocode for our approach. Details are available in our technical publication [22]

**Fig. 5:**
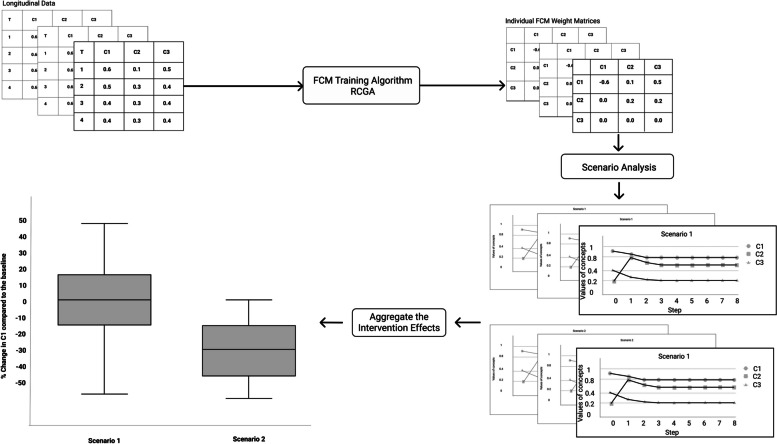
The data analysis process

Since an FCM serves to support decision making activities, its construction is generally followed by its use on scenarios of interest (Fig. 5). In our case, the scenarios consist of hypothetical interventions, whose results are estimated by running simulations on the FCM. Simulations were performed using the FCMpy package in Python [23]. In this section, we describe each step in more detail.

### Constructing a fuzzy cognitive map

#### Step 1: Identify the determinants in the problem domain

In the first step of constructing an FCM, the modeler must identify all the determinants (i.e., nodes) relevant to the problem domain. This can be achieved either by *directly* collecting relevant information from the stakeholders (e.g., domain experts, community members) or by obtaining such information *indirectly* from secondary sources (e.g., empirical papers, reports, archives, theories) [24, 25]. Indirect approaches can be applied when there are considerable constraints imposed on the project (e.g., hard to reach population, limited resources) or when the problem domain is well covered in secondary sources. A mixture of direct and indirect approaches is also possible in cases where the problem domain is partially covered in the literature [26]. In our case, Springvloet et al*.* [9] in their study have already identified 11 determinants in the empirical literature that are relevant to the problem domain. Some of these determinants constitute aggregate scores of several measurement items. For this study we selected determinants at the lowest possible level and therefore we considered the determinants at the level of measurement items that totals to 15 determinants (see Table 1).

This choice was guided by the notion that interventions operate on the determinants at the lowest level and thereby cannot directly target determinants on higher levels [27]. In the next step, we proceeded to the identification of causal weights between the determinants.

#### Step 2: Identify the causal weights (structure of the system)

In this step, we ought to identify causal links (and the associated weights) between the set of determinants listed in the previous step. These weights can be identified by experts, inferred from data, or a mix of both (when experts’ weights can be refined thanks to data). In our study, we learn the weights from data. While there are many evolutionary algorithms to perform this task, they share common characteristics: they begin with a random solution and aim to improve it by repeatedly applying operators such as *crossover*, *mutation*, and *selection* [28]. The quality of a solution at a given iteration is known as its *fitness*. In other words, the fitness measures the extent to which (through the links’ weights) an FCM can produce data that is “similar enough” to the real-world target data. Algorithmic solutions can differ in how they define the operators as well as their quantification of fitness. Solutions can be broadly categorized depending on whether they improve a single candidate solution or keep a pool of candidates at each step (known as a ‘population’). We use a population-based algorithm, which is the most prolific research area to create FCMs with over 20 algorithms proposed previously (see Table 1 in [29]).

Our algorithm modifies the foundational RCGA solution [12]. The RCGA computed the fitness as the difference between the final (simulated) outputs of an FCM and the real-world observed data. The RCGA was designed for data-scarce situations in which only the initial (baseline) and final value of a phenomenon were recorded. In our case, we have longitudinal measurements hence we know the values of concepts in each participant over several steps. We thus previously modified the RCGA [22] to ensure that the fitness was computed over each time step. This modification ensures that the optimized FCM closely follows the trajectory of an individual, instead of only replicating the final value.

The *crossover* is a way to mix two candidate solutions, seen as ‘parents’ from the perspective of evolutionary algorithms. For instance, we can cut each of two parents into two parts (i.e., split their matrices) and swap these entire parts, in a similar manner to how an offspring’s genome is obtained through a recombination of the parents’ genomes. In our case, the crossover consists of swapping the causal weight of an edge selected at random.

The *mutation* will replace some of the values. This is a way to introduce more variability in the pool of potential solutions. A mutation can be an advanced process, for example if we have reasons to control the level of variability (i.e., ensure that each offspring resembles its parent to a measurable level). In our case, in line with the original RCGA algorithm, we perform a uniform mutation such that the causal weight of a randomly chosen edge is randomly reassigned.

If any of the new solutions is satisfactory, the algorithm can end by providing the solution along with its fitness. Otherwise, the algorithm will select the solutions with the highest fitness and resume the process.

**Fig. 6:**
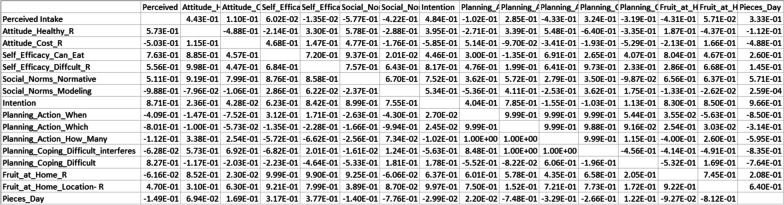
Example of an FCM produced by Genetic Algorithms. The FCM is shown as a matrix of weights, where concept names are the same between the x-axis and y-axis

Due to the randomness inherent to this algorithm, several FCMs can be provided (e.g., by running the algorithms a few times) that all provide a fitness that meets or exceeds the goal. This is illustrated in our third-party repository at https://osf.io/uj7wd/, under the archive ‘weights’, where we provided a comprehensive set of FCMs produced as the final product of our algorithm. An example of an FCM connection matrix produced by genetic algorithms is shown in Fig. 6.

The FCM connection matrices produced by our algorithmwere further evaluated by calculating the *in-sample* and *out of sample* errors. The *in-sample* error indicates the average difference between the observed data and the data from the simulation of the learned FCM using the same initial conditions. The *out of sample* error indicates the difference between the observed data and the data from the simulations using randomly generated initial conditions [21].

### Defining hypothetical intervention scenarios

Based on the developed logic model of the problem, Springvloet et al*.* [9] constructed two interventions (basic and plus). The *basic* intervention targeted only determinants within the domains of individual cognitions and self-regulation (i.e., awareness, attitude and self-efficacy). The *plus* intervention additionally provided environmental level feedback on availability and prices of fruit in the supermarket as well as on how to make fruit more available and accessible in the home environment. The intervention planners expected that either intervention would increase fruit intake among the participants compared to no treatment scenario.

To translate the above-described interventions (basic and plus) into the framework of an FCM, we needed to specify the determinants directly modified by these interventions [30] (i.e., intervention targets) and indicate the (hypothesized) effect of the interventions on these targets. To achieve this, we invited three experts from the Department of Health Promotion at Maastricht University who had a PhD in the field of public health or health promotion, were familiar with the context in the Netherlands (have at least one research report on the topic in the Netherlands), and had experience in the field of nutrition (at least two years of work experience). The experts were asked to assess the causal impact of the (hypothetical) interventions on the target determinants. In line with best practices for Fuzzy Cognitive Mapping, experts provided the causal impact using linguistic terms (very high, high, medium, low, very low and non-existent) rather than a Likert-scale.

**Fig. 7:**
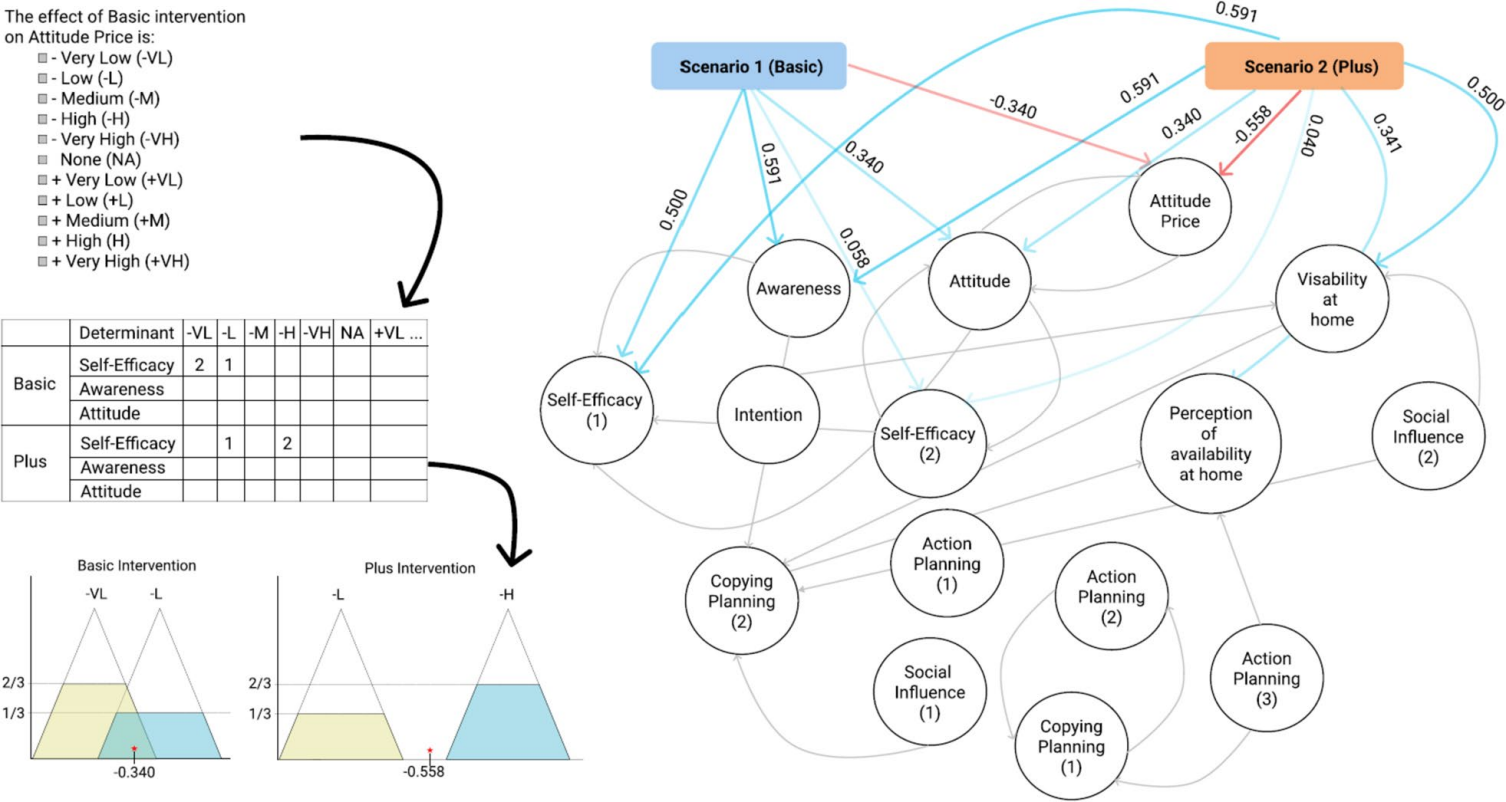
The process of computing causal impacts of interventions on the target determinants via fuzzy logic (the gray arrows are the causal weights between the determinants derived via RCGA)

After the experts evaluated the expected causal impact of the interventions on the target determinants by using linguistic terms, we applied fuzzy logic using the same four steps as in prior studies to obtain numerical causal weights [30–33]. First, we associated each of the linguistic terms with a triangular membership function [33–35]. For example, this can represent how an expert who says “medium” tends to mean 0.5 on a scale from 0 to 1 and there is a reduced (but non-zero) possibility for other values as we move away from this peak. In addition, these functions can overlap, which gives the possibility that experts with closely related terms (e.g., low and very low) have the same thought but expressed it differently. Second, for each causal relationship from an intervention to a determinant, we projected the experts’ responses onto the membership functions. For example, if we have three experts who said {Low, Medium, Medium} then 1/3 of the experts endorses ‘low’ and 2/3 of the experts endorse ‘medium’. We thus use each membership function to the extent in which it was endorsed by the experts ([36], which is known as ‘activation’; [33]). Third, we aggregated all the activated membership functions (with a Family Max aggregation operation) [37]. Lastly, we derived the final value for the intervention-determinant pair by using centroid defuzzification method [38]. By applying this procedure for each pair, we obtain a connection matrix that encodes information about the causal impact of the intervention on the target determinants (described in more detail in Mkhitaryan et al*.* [21]) (Fig. 7).

### Testing of intervention scenarios

Testing of intervention scenarios in an FCM framework is often implemented by either changing the baseline values of the determinants (single shot interventions) or by introducing the intervention as a new factor in the defined FCM that continuously influences the target determinants (continuous interventions) [28, 33]. In our case, we used the latter approach because the plus and basic interventions are designed in such a way that the participants are expected to be continuously exposed to the intervention.

After introducing the proposed interventions as part of the identified FCM structure, the effects of the interventions are examined by iteratively updating the values of the determinants by the following equation [39, 40]:

1 $${A}_{i}^{t+1}=f\left({A}_{i}+\sum\limits_{j=1}^{n}{A}_{j}^{t}*{W}_{ji}\right)$$

where ${A}_{i}$ is the value of concept $i$ at the simulation step $t$ and the ${W}_{ji}$ is the causal impact of concept $j$ on concept $i$. As can be noted in the equation two, a (transfer) function *f* is applied to the results to keep the concept values within a certain range. In our case, we chose a sigmoid function as the concept values should take values in the range of [0,1]. The sigmoid function can be expressed as [41]:

2 $$f\left(x\right)=\frac{1}{1+{e}^{-\lambda x}}$$

where $$\lambda$$ is a positive number that determines the steepness of the sigmoid function ($$0< \lambda \le 10$$). The concept values are updated until the change in either all or a subset of these values ($$|{A}_{i}^{t+1}-{A}_{i}^{t}|$$) is not more than a specified threshold (e.g., $$0.001)$$ or a specified maximum number of iterations is reached (e.g., 2000) [19]. In our case, we did not specify a subset of concepts and let the algorithm run until the change in all the concept values did not exceed the threshold.

## Results

The RCGA produced FCM connection matrices for each participant (257 matrices in total). The median in sample and out of sample errors of the weight matrices were 0.36 (SD 0.086) and 0.145 (SD = 0.023) respectively. The experts specified two hypothetical intervention scenarios (*basic* and *plus*) to be tested in an FCM framework. Based on the expert inputs, we derived the effects of the interventions on the target determinants (Table 2).

**Table 2:** Intervention scenarios and their causal impacts on the target determinants. Target determinants are described with short labels; see Table 1 for complete descriptions

Intervention Scenarios	Target Determinants						
	Awareness	Attitude	Attitude Price	Self-efficacy 1	Self-efficacy 2	Perception of availability at home	Visibility at home
Basic	0.591	0.340	-0.340	0.500	0.058	N/A	N/A
Plus	0.591	0.340	-0.558	0.591	0.040	0.341	0.500

The results of the simulations of the hypothetical intervention scenarios showed that both interventions would produce similar effects on the determinants of fruit intake, except for the visibility of fruits at home. More specifically, the *plus* intervention would have a stronger positive impact on the change in the mentioned determinants compared to the *basic* intervention (Table 3).

**Table 3:** Percent change in the determinants in the Basic and Plus interventions

	**% Change compared to the baseline (± SD)**	
**Determinant**	**Basic Intervention**	**Plus Intervention**
Awareness	30.453 (19.684)	30.841 (20.831)
Attitude	5.984 (5.68)	5.813 (5.594)
Attitude Price	-21.002 (8.705)	-32.493 (9.678)
Self-efficacy	15.772 (20.441)	13.558 (18.138)
	3.096 (7.988)	2.23 (8.407)
Social-influence	1.021 (6.634)	1.095 (7.563)
	1.055 (6.613)	1.154 (8.313)
Intention	0.152 (6.194)	0.206 (6.915)
Action-planning	0.142 (9.744)	-0.298 (11.383)
	-1.329 (8.423)	-1.509 (10.39)
	-0.749 (7.925)	-0.672 (9.46)
Copying planning	0.192 (8.797)	0.207 (10.341)
	1.379 (10.818)	1.843 (12.413)
Perception of availability at home	1.379 (10.818)	2.98 (5.203)
Visibility at home	-0.143 (3.655)	4.372 (11.325)

**Fig. 8:**
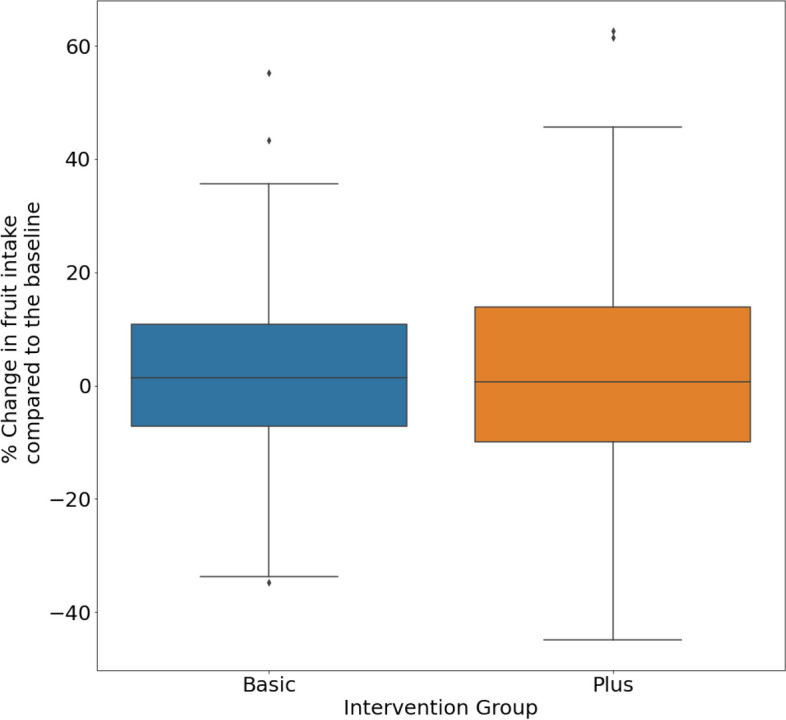
Percent change in fruit intake at group level compared to baseline in Basic and Plus intervention scenarios

Furthermore, the simulations showed that the average effect (percent change compared to the baseline) of both interventions on fruit intake across all participants would yield similar results (mean difference in the percent change in fruit intake compared to the baseline 1.86% and 2.43% respectively) (Fig. 8).

**Fig. 9:**
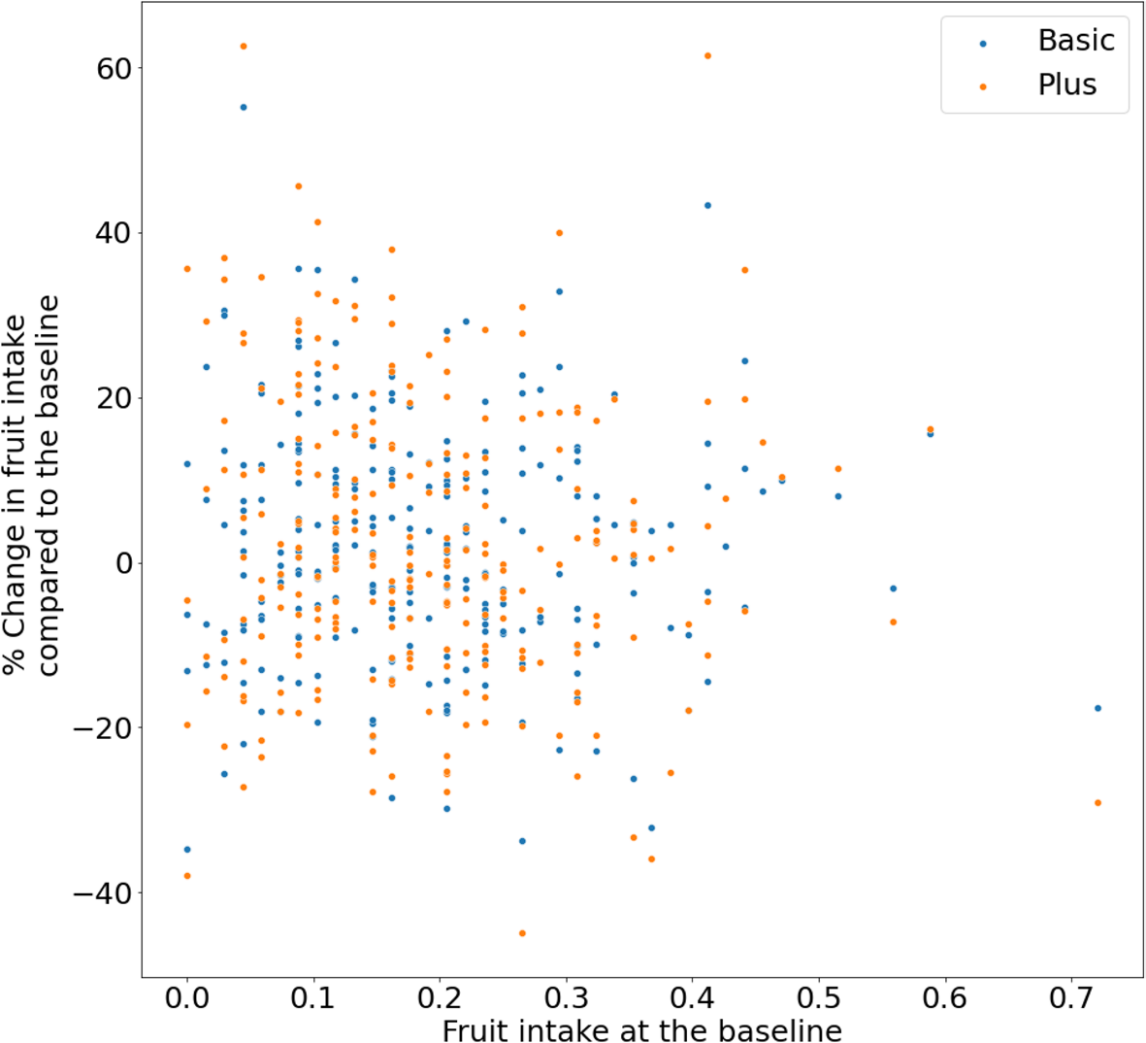
Percent change in fruit intake at individual level after an intervention compared to baseline

The effect of the interventions on the change in fruit intake did not seem to depend on the levels of fruit intake at baseline (Fig. 9).

## Discussion

Intervention planners use logic models to design evidence-based interventions. Logic models are the backbone of the theories of change which encapsulate information on how interventions are *expected* to impact the target variable/s. Logic models can vary in their complexity and the more complex the logic models become the more they necessitate the use of additional computational techniques to guide decisions. Indeed, logic models that incorporate complex structures (feedback loops) need to be simulated until loops have stabilized, instead of simple chains of lists in which effects can be manually tracked on a diagram.

In the present paper, we demonstrated how machine learning can be used with FCMs to assist in developing health behavior interventions that accounted for such complex structures. In this framework and in line with previous studies, machine learning allows building FCMs relying on historical data without imposing any restriction on the structure of the system (e.g. loops are allowed) [21, 42]. In our study, we showed that it was even possible to generate a variety of FCMs that each correspond to an individual, thus evaluating the potential effect of interventions across a population.

The simulation engine of the FCMs allows testing hypothetical intervention scenarios. By doing so, the intervention planners have the opportunity to finetune the intervention components thus increasing its potential effectiveness. For demonstration purposes, the two scenarios used in this study were based on the scenarios that were actually tested in real-life in the evaluation study. This allows for comparison of the results of the simulations with the real-life evaluation study. For example, the results of the FCM simulations of the intervention cases presented in this paper showed that the effect found in the two intervention scenarios (basic and plus) would be very small. This finding is consistent with the results of the evaluation study of the intervention we used as a case study [12], thus providing external validity to the approach. The evaluation study of the real life intervention found a small difference between the two interventions at four months follow-up (plus vs basic: *d* = 0.22, *P* = 0.04) [12]. The presented approach uses a single case study in the context of nutrition within the Netherlands, so the validity of the approach could be strengthened by complementary studies on different populations and different endpoints/behaviors. Additionally, the reliability of the hypothetical interventions could be further strengthened by involving more domain experts.

The demonstrated approach can also be used to test multiple intervention scenarios that cannot all be tested in real-life (e.g., due to constraints in resources, such as budget and time). Additionally, it can be used to test multiple intervention scenarios and use the results to make an informed decision regarding which scenarios to test in a real-life evaluation study.

Building an FCM for health behavior interventions is a challenging task. The challenge lies in the paucity of the theoretical and empirical knowledge-base on the causal relationships between the determinants at lower levels. To build an FCM for health behavior interventions, we need to have sufficient knowledge on the mechanics by which various determinants impact one another. Such knowledge is essential for FCMs that are entirely based on expert knowledge (i.e., expert-based FCMs), quantitative data (e.g., data driven approaches) or a combination of both (i.e., hybrid approaches). Our study demonstrates a potential combination, by using a data-driven approach to create an FCM for each individual based on their empirical data, while involving subject-matter experts to qualitative assess the expected strength of an intervention.

Although our method was able to quickly provide satisfactory individual FCMs for a case study of hundreds of adults and a few measurements, it is possible that intervention planners either have access to a much larger population or use a significant larger number of measurements. In these cases, scalability with respect to population size and/or measurements become important considerations. In a recent study [43], we showed that the Genetic Algorithms used in our process (Algorithm 1) can be improved by using a state-of-the-art optimization algorithm known as CMA-ES (Covariance Matrix Adaptation Evolution Strategy) instead of manually performing core operations such as crossover, mutation, and selection. As a result, we achieve a 15 × speed-up and our approach can be extended to large populations or many measurements while continuing to provide high fidelity (with respect to individual trajectories of behavior change) and delivering results within a satisfactory timeframe. The timeframe scales linearly with respect to the number of individuals (i.e., if an intervention planner has twice the population size then it will take twice as long) and the growth is sublinear with respect to the number of measurements (i.e., if an individual is defined by twice the number of steps then it takes under twice the amount of time).

While our study used Fuzzy Cognitive Mapping to capture the dynamics of health behaviors, there are other techniques which also produce simulation models in the form of networks. For instance, System Dynamics (SD) has been abundantly used for health behaviors [44–46] and this technique also structures a model through concepts connected by edges. In addition, Genetic Algorithms have also been used to calibrate SD models [47]. However, FCM and SD are employed in different situations. As explained in our Background section, FCMs are appropriate when data is partly qualitative, for example if data originates from surveys in which fuzzy qualifiers are used (e.g., ‘very strong’, ‘medium’) [48]. In contrast, SD is the tool of choice when data is quantitative so that edges are expressed as rates (i.e., units of flow for a given time window). Our method is thus most applicable when health behaviors have been tracked through surveys rather than when precise measurements have been obtained for each construct over time.

To fully leverage the potential of the FCM framework for health behavior interventions, future work should be dedicated to consolidating theories and empirical evidence on the relationships between the determinants at lower levels. Furthermore, the machine learning algorithms for FCMs should be extended to allow imposing of constraints on the FCM structure to produce FCMs that are consistent with available theories and the empirical evidence [49].

## Data Availability

The datasets used and/or analyzed during the current study are available from the corresponding author on reasonable request.

## Abbreviations

FCM: Fuzzy Cognitive Maps
RCGA: Real Coded Genetic Algorithm
